# Targeting DNA Replication and Repair for the Development of Novel Therapeutics against Tuberculosis

**DOI:** 10.3389/fmolb.2017.00075

**Published:** 2017-11-14

**Authors:** Michael A. Reiche, Digby F. Warner, Valerie Mizrahi

**Affiliations:** SAMRC/NHLS/UCT Molecular Mycobacteriology Research Unit, DST/NRF Centre of Excellence for Biomedical Tuberculosis Research, Department of Pathology, Institute of Infectious Disease and Molecular Medicine, Faculty of Health Sciences, University of Cape Town, Cape Town, South Africa

**Keywords:** DNA replication, Tuberculosis, bacteria, drug resistance, drug targets

## Abstract

*Mycobacterium tuberculosis* is the etiological agent of tuberculosis (TB), an infectious disease which results in approximately 10 million incident cases and 1.4 million deaths globally each year, making it the leading cause of mortality from infection. An effective frontline combination chemotherapy exists for TB; however, this regimen requires the administration of four drugs in a 2 month long intensive phase followed by a continuation phase of a further 4 months with two of the original drugs, and is only effective for the treatment of drug-sensitive TB. The emergence and global spread of multidrug-resistant (MDR) as well as extensively drug-resistant (XDR) strains of *M. tuberculosis*, and the complications posed by co-infection with the human immunodeficiency virus (HIV) and other co-morbidities such as diabetes, have prompted urgent efforts to develop shorter regimens comprising new compounds with novel mechanisms of action. This demands that researchers re-visit cellular pathways and functions that are essential to *M. tuberculosis* survival and replication in the host but which are inadequately represented amongst the targets of current anti-mycobacterial agents. Here, we consider the DNA replication and repair machinery as a source of new targets for anti-TB drug development. Like most bacteria, *M. tuberculosis* encodes a complex array of proteins which ensure faithful and accurate replication and repair of the chromosomal DNA. Many of these are essential; so, too, are enzymes in the ancillary pathways of nucleotide biosynthesis, salvage, and re-cycling, suggesting the potential to inhibit replication and repair functions at multiple stages. To this end, we provide an update on the state of chemotherapeutic inhibition of DNA synthesis and related pathways in *M. tuberculosis*. Given the established links between genotoxicity and mutagenesis, we also consider the potential implications of targeting DNA metabolic pathways implicated in the development of drug resistance in *M. tuberculosis*, an organism which is unusual in relying exclusively on *de novo* mutations and chromosomal rearrangements for evolution, including the acquisition of drug resistance. In that context, we conclude by discussing the feasibility of targeting mutagenic pathways in an ancillary, “anti-evolution” strategy aimed at protecting existing and future TB drugs.

## Introduction

### The need for new TB drugs

According to the most recent WHO report, 10.4 million people developed tuberculosis (TB) and 1.8 million died from this disease in 2015 (WHO, 2016), making TB the leading cause of death from an infectious disease. The threat that TB presents to global health has been significantly heightened by the evolution and spread of drug-resistant TB: in 2015, a staggering 480,000 people across the world developed multi-drug resistant (MDR)-TB, defined as TB that is resistant to isoniazid (INH) and rifampicin (RIF), with or without resistance to other first-line anti-tubercular drugs. Of these, 9.5% had extensively drug-resistant (XDR)-TB, which is resistant to INH and RIF (i.e., MDR-TB) in addition to any fluoroquinolone and at least one of the injectable second-line drugs, kanamycin, amikacin, or capreomycin. Unfortunately, this alarming situation has continued to worsen with the ongoing evolution of XDR-TB to forms of the disease that are functionally untreatable with existing antibiotics (Dheda et al., 2014).

Drug-sensitive TB is treated with a standard “short-course” regimen comprising a 2-month intensive phase of treatment with four drugs—INH, RIF, pyrazinamide (PZA), and ethambutol (EMB)—followed by four additional months of treatment with INH and RIF in a continuation phase. Under optimal conditions, this regimen is highly effective at achieving durable cure of drug-sensitive TB. However, non-adherence to this protracted therapeutic regimen is common among TB patients and may result in the emergence of drug resistance through the acquisition of chromosomal mutations in the aetiological agent, *Mycobacterium tuberculosis* (*M. tuberculosis*), leading to prolonged infectiousness and poor treatment outcomes (Dheda et al., 2014). Drug-resistant TB is far more challenging to treat, requiring the administration of combinations of second- and third-line drugs that are more toxic, more expensive, and less efficacious. As a result, this form of the disease is associated with substantial morbidity and mortality, while consuming a disproportionate share of national budgets for TB control in disease-endemic countries—thus compromising TB control programmes (Dheda et al., 2014, 2017).

The need for new TB drugs for the treatment of drug-susceptible as well as drug-resistant TB is therefore clear and urgent. After decades of neglect, a renewed interest in TB drug development in the late 1990s, which coincided with major scientific advances including the completion of the first genome sequence of *M. tuberculosis* (Cole et al., 1998), has resulted in a pipeline populated with new as well as repurposed drugs and drug combinations at various stages of development (http://www.newtbdrugs.org). A number of criteria are being used to guide this process: for example, all new TB drugs should: (i) have novel mechanisms of action to permit their use in the treatment of drug-resistant forms of the disease; (ii) have significant treatment-shortening potential when combined with other agents; (iii) be safe and tolerable; (iv) simplify treatment by reducing the pill burden and dosing frequency; and, (v) be compatible with antiretroviral drugs to enable treatment of patients co-infected with HIV (Zumla et al., 2013, 2014). The ability to meet these criteria is dependent upon the quality of compounds that enter the pipeline at the lead optimization stage. The identification of high-quality leads has, in turn, been critically reliant on harnessing biological insight from studies on *M. tuberculosis* pathogenesis in various models of infection. A major theme emerging from this work is the biological complexity of TB at the level of both host and pathogen, with the genotypic and phenotypic heterogeneity of *M. tuberculosis* posing particularly onerous challenges for new TB drug discovery, as discussed below.

### Approaches to TB drug discovery

Genome-wide mutagenesis studies in *M. tuberculosis* (Long et al., 2015) have identified genes that are (conditionally) essential for growth and survival of the bacillus *in vitro* (Sassetti et al., 2003; DeJesus et al., 2017), in macrophages (Rengarajan et al., 2005), and in animal models of infection (Sassetti and Rubin, 2003). This information has underpinned target-based drug discovery efforts aimed at crippling essential cellular functions in *M. tuberculosis*. However, as in other areas of antimicrobial drug discovery (Payne et al., 2007), the approach has met with very limited success in the TB field, and has been confounded by a general lack of information about target vulnerability as well as the impact of compound metabolism, permeability, and efflux on efficacy. For this reason, small molecules that potently inhibit *M. tuberculosis* enzymes in biochemical assays have failed to translate into leads with activity against the bacillus *in vitro* and/or *in vivo*. In contrast, phenotypic screening, in which compound libraries are screened for activity against *M. tuberculosis* to identify molecules with whole-cell activity, has been far more successful, and has delivered the clinically approved drugs, bedaquiline (Sirturo) and delamanid (Deltyba), a number of drug candidates that are currently in development (Mdluli et al., 2015; Singh and Mizrahi, 2017)—including griselimycin (Kling et al., 2015), PA-824 (pretomanid) (Stover et al., 2000), PBTZ169 (Makarov et al., 2014), and Q203 (Pethe et al., 2013)—and other promising leads such as the Pks13 inhibitor, TAM16 (Aggarwal et al., 2017). It is worth noting, however, that this approach, too, has its challenges as mechanisms of action (MOA) of potent molecules with whole-cell activity can be difficult to elucidate, thereby complicating the progression of individual compounds or compound series through the pipeline. Importantly, though, there are signs indicating greater integration of the two approaches: on the one hand, target-based whole-cell screening, in which hit identification from phenotypic screening is biased toward prioritized targets and pathways, has begun to gain traction (Abrahams et al., 2012) while, on the other hand, screening collections of whole-cell actives identified by phenotypic approaches against high-value *M. tuberculosis* targets offers the prospect of discovering new drug-target pairs as starting points for hit-to-lead (H2L) programs (Esposito et al., 2017).

### Managing biological complexity in TB drug discovery

Genotypic and phenotypic heterogeneity of *M. tuberculosis* must be taken into account from the earliest stage of TB drug discovery. Genotypic heterogeneity is managed by screening promising hits for activity against representatives from the major strain lineages of *M. tuberculosis* (Coscolla and Gagneux, 2014) and against panels of drug-resistant strains (e.g., Aggarwal et al., 2017; Blondiaux et al., 2017). The other major mechanism underlying differential drug susceptibility in *M. tuberculosis* is phenotypic antibiotic tolerance (Aldridge et al., 2014; Brauner et al., 2016), which is thought to be the main reason why prolonged TB therapy is required in order to achieve relapse-free cure (Kester and Fortune, 2014; Gold and Nathan, 2017). Antibiotic efficacy can be influenced profoundly by the physiology, metabolic state, and growth rate of the organism, with most TB drugs showing significantly reduced efficacy against *M. tuberculosis* in slow- or non-growing states (Baer et al., 2015). Thus, drugs that target cellular processes required to support bacterial growth tend to have reduced efficacy against slow- or non-growing organisms (Gold and Nathan, 2017). *Mycobacterium tuberculosis* encounters complex, hostile environments during transmission, infection, and disease (Pai et al., 2016). As an exquisitely adapted human pathogen endowed with a rich and highly flexible metabolic repertoire (Baughn and Rhee, 2014; Warner, 2014), the bacillus is able to adapt its physiology and metabolism in response to the conditions encountered during each of these stages. These conditions include intracellular residence in macrophages and other phagocytic cells, exposure to nitrosative and oxidative stress, hypoxia, nutrient deprivation, alterations in carbon source availabilities, and low pH (Baer et al., 2015). In a single patient, therefore, *M. tuberculosis* infection can be characterized by mixed populations of intracellular and extracellular bacilli in a variety of metabolic states and with variable growth rates. This complicates treatment (Dartois and Barry, 2013) and has led to the suggestion that TB should be treated as a polybacterial infection (Evangelopoulos and McHugh, 2015). The problem is further complicated by the impact of lesion heterogeneity on drug pharmacokinetic/pharmacodynamic (PK/PD) parameters (Dartois, 2014). To address this complexity, assays designed to recapitulate at least some of the conditions encountered during infection have been incorporated into drug screening cascades with the aim of identifying “pan-active” compounds with the ability to kill *M. tuberculosis* in as wide a range of metabolic states as possible.

### Major mechanistic classes of TB drugs

TB drugs fall into a relatively small number of mechanistic classes. A defining characteristic of the tubercle bacillus is its unusual and highly complex cell envelope, which has a number of distinguishing features including the mycolyl-arabinogalactan-peptidoglycan complex that links the peptidoglycan to the mycobacterial outer membrane. Not surprisingly, a disproportionate number of TB drugs act on biogenesis of the cell envelope; these include INH and ethambutol (EMB), the second-line agent, D-cycloserine, and those that act on the new targets, DprE1 (e.g., PBTZ169) (Makarov et al., 2014), MmpL3 (e.g., BM212 and other chemotypes) (Xu et al., 2017), and Pks13 (TAM16) (Aggarwal et al., 2017). Other drugs target transcription (RIF), protein synthesis (e.g., linezolid), and energy metabolism (bedaquiline, Q203). Furthermore, and consistent with the formidable capacity of *M. tuberculosis* to metabolize xenobiotics (Awasthi and Freundlich, 2017), prodrugs are common in the TB drug arsenal and, for compounds such as PZA, delamanid, and pretomanid, the respective active metabolites have pleiotropic effects on mycobacterial metabolism (Matsumoto et al., 2006; Singh et al., 2008; Anthony et al., 2016).

An important, albeit small, category of TB drugs includes those that target DNA replication. Until recently, these have been limited exclusively to the fluoroquinolones, in particular, moxifloxacin and gatifloxacin, which inhibit DNA gyrase, and are widely used for the treatment of MDR-TB. However, another component of the DNA replication machinery has emerged as an exciting new target for TB drug development through the discovery that griselimycins target the β-clamp protein, DnaN (Kling et al., 2015). In the following sections, we consider the DNA replication and repair pathways of *M. tuberculosis* as potential sources of new targets for TB drug development. This terrain has been extensively reviewed recently, perhaps signaling the increasing appreciation of DNA metabolism as underrepresented among common antibiotic targets. The interested reader is encouraged to consult a number of excellent articles, both specific to *M. tuberculosis* (Plocinska et al., 2017) and of more general interest (Robinson et al., 2012; Sanyal and Doig, 2012; van Eijk et al., 2017).

## The mycobacterial DNA replication machinery

Chromosomal replication in bacteria is performed by a large, multiprotein replisome that ensures coordinated synthesis of the leading and lagging DNA strands with high efficiency and accuracy (Beattie and Reyes-Lamothe, 2015; Yao and O'Donnell, 2016a,b). Broadly, this is accomplished through the concerted action of three catalytic centers: the helicase-primase complex, the core complex, and the clamp loader complex [for comprehensive recent reviews, please refer to Ditse et al. (2017) and Plocinska et al. (2017). The helicase-primase complex comprises the DnaB helicase, which unwinds the two DNA strands, and the DnaG primase, which synthesizes short RNA primers on the lagging strand to initiate replication by the replicative DNA polymerase, Pol IIIα. Two core complexes containing Pol IIIα, the exonuclease subunit, ε, and the small subunit, θ, synthesize the new DNA strand on both leading and lagging strand templates. In elegant *in vivo* studies that were directed by earlier *in vitro* studies by Yao and O'Donnell (Yao and O'Donnell, 2016a,b) and the identification of the β-clamp (O'Donnell and Kuriyan, 2006), Reyes-Lamothe and colleagues demonstrated that these core complexes bind to the torroidal β-clamp proteins that encircle the DNA, providing a tether that enables processive synthesis and dynamic exchange of replisome components (Reyes-Lamothe et al., 2012). A $\tau_{3}\delta_{1}\delta^{\prime }$_1_χ_1_ψ_1_ clamp-loader complex loads the β-clamp proteins onto newly synthesized RNA primers, with the τ subunits also binding to the Pol IIIα subunits to couple leading and lagging strand biosynthesis, and the χ/ψ subunits guiding single-stranded DNA binding (SSB) proteins onto the DNA lagging strand.

The composition of the replisome is dynamic (Beattie et al., 2017; Lewis et al., 2017) and, as evident from the brief description above, the majority of the constituent proteins perform specialist functions ranging across DNA unwinding, RNA primer synthesis, clamp loading, and DNA synthesis. It is not surprising, therefore, that most of the replisome components are conserved across bacteria (Robinson et al., 2012), including *M. tuberculosis* (Ditse et al., 2017). So, while replisome function has been most thoroughly investigated in organisms such as *E. coli* and *B. subtilis* (Beattie and Reyes-Lamothe, 2015), the resulting models of the bacterial replication machinery are considered readily applicable to less studied systems, such as *M. tuberculosis*, with some notable exceptions (Ditse et al., 2017). For example, there are no clear homologs of several initiation proteins (DnaC, DnaT, PriB, and PriC) in *M. tuberculosis*, neither is there a *holE*-encoded θ subunit, nor *holC*- and *holD*-encoded χ and ψ clamp-loader subunits, respectively. Moreover, recent studies have revealed additional departures of the mycobacterial system from the classic replication models, most notably in demonstrating a dominant role for the PHP domain of the essential Pol IIIα subunit, DnaE1, in proofreading in *M. tuberculosis* (Rock et al., 2015; Gu et al., 2016), as discussed elsewhere (Ditse et al., 2017).

### Targeting DNA replication in *M. tuberculosis*

The *M. tuberculosis* genome comprises approximately 3950 genes (Cole et al., 1998; Wang and Chen, 2013), of which ~10% (461 genes) are absolutely required for growth and survival of the bacillus under standard aerobic growth conditions *in vitro* (DeJesus et al., 2017). Among the “essential” genes, 15 encode components of the DNA replication machinery; these include the DnaA replication initiator, PriA helicase loader, DnaB helicase, DnaG primase, SSB, clamp loader subunits (τ/γ, δ, δ′), DNA polymerases I and III, DnaN β-clamp, DNA ligase I, and type I and II topoisomerases (Ditse et al., 2017). It is notable that effective inhibitory agents are available for only a small number of these essential mycobacterial proteins (Table 1), with DNA gyrase representing the only clinically validated target—of the fluoroquinolones, which are used in treatment of MDR-TB. This implies considerable scope for developing new compounds targeting the other essential DNA replication components, as has been proposed recently for *M. tuberculosis* as well as other bacterial pathogens (Robinson et al., 2012; Sanyal and Doig, 2012; Plocinska et al., 2017; van Eijk et al., 2017). In turn, it also suggests the possible utility in investigating the potential antimycobacterial efficacies of compounds developed for use against homologous DNA replication and repair proteins in other bacteria (Table 2).

**Table 1:** Essential proteins involved in DNA replication targeted by anti-tubercular compounds.

**Name^a^**	***In vitro* essentiality**	**Inhibitor or compound series**	**Target IC_50_ (μM)**	**MIC (μM)**	**References**
DnaN/β (Rv0002)	Essential^1^^,^^3^	Griselimycins		0.05–0.84	Kling et al., 2015
GyrB (Rv0005)	Essential^1^^,^^2^	Novobiocin	1	6.5	Chopra et al., 2012
		Pyrrolamides	<0.5	0.026–1.7	Hameed et al., 2014
		Thiazolopyridine		0.0005	Kale et al., 2013
		Aminopyrazinamides	<0.002–>50	<1.0–>81	Shirude et al., 2013
		Thiazole-aminopiperidine hybrid analogs	50	28.44	Jeankumar et al., 2013
		Methoxyquinolone carboxylic acids	>102.96	0.16–6.43	Senthilkumar et al., 2008
		Benzothiazinone-piperazine derivatives	0.51-26	1.82–52	Chandran et al., 2015
		*N*-linked aminopiperidines	>3.6	6.2–132	Jeankumar et al., 2014
		Benzofurans	0.81		Renuka et al., 2014
			0.42		Reddy et al., 2014
		Quinoxalines and quinoxaline analogs	12–50		Sipos et al., 2015
		Phenylthiophene carboxamides	>0.76	4.84–78.5	Saxena et al., 2015
		Quinoline–aminopiperidine hybrid analogs	0.62–34.5	1.72–67.94	Medapi et al., 2015a
		7-Methyljuglone	30	2.6	Karkare et al., 2013
		Diospyrin	15	21.4	Karkare et al., 2013
		Indoline-dione Schiff bases	>40		Aboul-Fadl et al., 2011
		4-Aminoquinoline derivatives	0.63–23.92	1.47–49.75	Medapi et al., 2015b
		Thiazolopyridone ureas		0.2–19	Kale et al., 2013, 2014
		7-chloroquinolinyl-piperazinyl-pyridinylmethyl acetamide derivatives	1.82–28.3	7.26–76.55	Jeankumar et al., 2016a
		Benzo-imidazolyl acid derivatives	0.5–25	7.2–64.14	Jeankumar et al., 2016b
		VXc-486		0.28–0.58	Locher et al., 2015
		7-substituted-naphthyridinone derivatives^#^		0.02–0.65	Blanco et al., 2015
GyrA (Rv0006)	Essential^1^^,^^2^	Moxifloxacin^#^	11.2	0.31–2.49	Aubry et al., 2004; Sulochana et al., 2005
		Gatifloxacin^#^	7.99	82.58–319.7	Alvirez-Freites et al., 2002; Aubry et al., 2004
		Ofloxacin derivatives^#^	>10	0.47–10	Dinakaran et al., 2008
		Gatifloxacin derivatives^#^	8–26.6	0.033–2.1	Sriram et al., 2006
		Fluoroquinolone DC-159a^#^		0.143	Disratthakit and Doi, 2010
		Acridine derivatives	5.21–33.9	6.46–57.80	Medapi et al., 2016
DnaE1/α (Rv1547)	Essential^1^^,^^2^	251D			Butler et al., 2007; Chhabra et al., 2011
DnaG (Rv2343c)	Essential^1^^,^^2^	Doxyrubicin (anthracyclines)	100		Kuron et al., 2014; Gajadeera et al., 2015
		Aloe-emodin			Gajadeera et al., 2015
LigA (Rv3014c)	Essential^1^^,^^2^	Bis-xylofuranosylated diamines	11.4–260		Srivastava et al., 2005a,b
		*N*-substituted tetracyclic indoles	13.5		Srivastava et al., 2007
		Pinafide and Mitonafid	>50	>25	Korycka-Machala et al., 2017
		Pyridochromanone	0.6		Gong et al., 2004
TopA/Top I (Rv3646c)	Essential^1^^,^^2^	Polyamine scaffolds	5-15		Sandhaus et al., 2016
		Hydroxycamptothecin derivatives	>2.9	5.46–48.36	Sridevi et al., 2015
		Amsacrine and Tryptanthrin	15-42		Sridevi et al., 2015
		m-AMSA		125	Godbole et al., 2014
		Norclomipramine and Imipramin		60–250	Godbole et al., 2015
		Dihydrobenzofuranyl urea	60		Ravishankar et al., 2015

a Cole et al. (1998),

1 DeJesus et al. (2017),

2 Griffin et al. (2011),

3 Xu et al. (2014),

# *Elucidation of the targets of DNA gyrase inhibitors is often complex and involves both GyrA and GyrB subunits*.

**Table 2:** Compounds worth investigating that inhibit homologs of essential *Mtb* proteins validated in other bacterial species^a^.

***Mtb* homologue^b^**	**Annotated function^b^**	***In vitro* essentiality**	**Inhibitor or compound series**	**Organism**	**References**
DnaA (Rv0001)	Initiation of DNA replication	Essential^1^^,^^2^	3-acetoxy-bi-indols	*E. coli*	Mizushima et al., 1996
			Sporulation protein SirA	*B. subtilis*	Rahn-Lee et al., 2011
DnaN (Rv0002)	β subunit of DNA polymerase III	Essential^1^^,^^3^	Small-molecule RU7	*S. pyogenes*; *E. coli*	Georgescu et al., 2008
			Toxin-antitoxin SocB	*C. crescentus*	Aakre et al., 2013
GyrB (Rv0005)	DNA gyrase, subunit B	Essential^1^^,^^2^	Spiropyrimidinetriones	Various Gram-negative and Gram-positive bacteria	Basarab et al., 2014, 2015
			Quinoline pyrimidine triones	Various Gram-negative and Gram-positive bacteria	Miller et al., 2008
			Isothiazolopyridones	*E. coli*; *S. aureus*	Wiles et al., 2006a
			Isothiazoloquinolones	*E. coli*; *S. aureus*	Wiles et al., 2006b
			3-amino quinazolinediones	Various Gram-negative and Gram-positive bacteria	Tran et al., 2007; Hutchings et al., 2008
			Cyclothialidines	Various Gram-positive bacteria	Angehrn et al., 2004, 2011; Lubbers et al., 2007
			Benzothiazole ethyl urea inhibitors	Various Gram-negative and Gram-positive bacteria	Stokes et al., 2013
			Tricyclic pyrrolopyramidine derivatives	Various Gram-negative and Gram-positive bacteria	Tari et al., 2013a,b
			Indazole derivatives	*S. aureus*; *S. pneumoniae*; *E. faecium*; *E. faecalis*	Zhang et al., 2015
			Benzimidazole ureas	*S. aureus*; *E. faecium*; *S. pneumoniae*; *E. faecalis*	Grillot et al., 2014
GyrA (Rv0006)	DNA gyrase, subunit A	Essential^1^^,^^2^	Simocyclinone D8	*E. coli*	Flatman et al., 2005
			Novel bacterial topoisomerase inhibitors	*S. aureus*; *E. coli*	Bax et al., 2010
			NXL101	Gram-positive bacteria	Black et al., 2008
SSB (Rv0054)	Helix-destabilizing protein	Essential^1^^,^^3^	Small-molecule inhibitors	*K. pneumonia*	Voter et al., 2017
			SSBA inhibitors	*E. coli; S. aureus; B. anthracis; F. tularensis*	Glanzer et al., 2016
DnaB (Rv0058)	DNA helicase	Essential^1^^,^^2^	Coumarin scaffolds	Gram-positive bacteria	Aiello et al., 2009; Li et al., 2012, 2013
			Flavonols	*E. coli*	Griep et al., 2007
				*K. pneumoniae*	Chen and Huang, 2011; Lin and Huang, 2012
			Triaminotriazines	*S. aureus*	McKay et al., 2006
PriA (Rv1402)	Primosomal helicase	Essential^1^	Kaempferol	*S. aureus*	Huang et al., 2015
			Small-molecule inhibitors	*K. pneumonia*	Voter et al., 2017
DnaE1 (Rv1547)	DNA polymerase III α	Essential^1^^,^^2^	Nargenicin	*S. aureus*; *E. coli*	Painter et al., 2015
			6-anilino-pyrimidine-diones	*B. subtilis*	Tarantino et al., 1999a
			Substituted deazaguanines	*B. subtilis*; *S. aureus*	Xu et al., 2011
DnaG (Rv2343c)	Primase	Essential^1^^,^^2^	Phenolic monosaccharides	*E. coli*	Hegde et al., 2004
			(p)ppGpp	*E. coli*; *B. subtilis*	Maciąg et al., 2010
			Bicyclic macrolide	*E. coli*	Chu et al., 2003
			Pyrido-thieno-pyrimidines	*E. coli*	Agarwal et al., 2007
			Benzo-pyrimido-furans	*E. coli*	Agarwal et al., 2007
LigA (Rv3014c)	DNA ligase	Essential^1^^,^^2^	6-azaindazoles	Gram-positive bacteria	Howard et al., 2013
			Pyridochromanones	*S. aureus*; *E. coli; S. pneumoniae; B. subtilis*	Brotz-Oesterhelt et al., 2003
			Arylamino compounds	*E. coli*; *S. typhimurium*	Ciarrocchi et al., 1999
			Adenosine analogs	Variety of Gram-negative and positive bacteria	Mills et al., 2011; Stokes et al., 2011
			Diamino-dimethylamino-pyrimido-pyrimidine	*S. pneumoniae*; *S. aureus*; *H. influenzae*	Meier et al., 2008
			Aminoalkoxypyrimidine carboxamides	*S. aureus*	Gu et al., 2012
			2-amino-naphthyridine-carboxamides	*S. aureus*; *S. pneumoniae*; *H. influenzae*	Surivet et al., 2012
			4-aminopyrido-pyrimidin-ones	*S. aureus*; *S. pneumoniae*; *H. influenzae*	Wang et al., 2012
			Adenine-based inhibitors	*S. pneumoniae; H. influenzae*	Buurman et al., 2012

*B. anthracis, Bacillus anthracis; B. subtilis, Bacillus subtilis; C. crescentus, Caulobacter crescentus; E. faecium, Enterococcus faecium; E. faecalis, Enterococcus faecalis; E. coli, Escherichia coli; F. tularensis, Francisella tularensis; H. influenzae, Haemophilus influenzae; H. pylori, Helicobacter pylori; K. pneumoniae, Klebsiella pneumoniae; S. aureus, Staphylococcus aureus; S. pneumonia, Streptococcus pneumoniae; S. pyogenes, Streptococcus pyogenes; S. typhimurium, Salmonella typhimurium*.

a Inhibition of either purified protein or bacterial growth;

b Cole et al. (1998);

1 DeJesus et al. (2017),

2 Griffin et al. (2011), and

3 *Xu et al. (2014)*.

As applies to antibiotic development in general, overcoming the natural defenses of the target organism—in particular, the permeability barrier presented by the (myco)bacterial cell wall, and the capacity for xenobiotic extrusion via multiple efflux pumps—is often a key challenge, particularly in converting hits from biochemical assays into whole-cell actives. Avoiding compound metabolism (degradation or modification) by the target bacillus or its human host can present an additional obstacle. For DNA replication and repair specifically, the non-availability to date of purified forms of many of the mycobacterial proteins and/or reconstituted complexes has further restricted the number of *in vitro* screens against purified proteins, and has required that researchers rely on homology models developed using template structures from other bacteria. Importantly, this can also complicate any assessment of the druggability and ligandability of the target protein—both key additional factors in determining the success of the antibiotic development process, and which render gene essentiality alone insufficient for target validation (Hopkins and Groom, 2002; Edfeldt et al., 2011). It is pleasing to note, therefore, that several recent successes in expressing different components of the mycobacterial DNA metabolic machinery (Gong et al., 2004; Rock et al., 2015; Gu et al., 2016; Banos-Mateos et al., 2017) suggest this critical roadblock will be overcome shortly.

Targeting DNA and the array of proteins which ensure its replication and maintenance within the cell presents an additional challenge, namely ensuring specificity of the applied drug for its target organism. This can be onerous given that the proteins which interact with and modify this macromolecule have retained many key features and commonalities as they have evolved in different species. For TB, which requires lengthy treatment, the need to avoid toxicity in the human host presents an additional major challenge, and one which is likely to exclude drugs which target DNA directly, such as DNA intercalating agents (Zhang et al., 2017), and inducers of replication stress in mammalian cells (i.e., anticancer compounds). Instead, antitubercular chemotherapies need to be designed to exploit specific nuances of, and vulnerabilities within, the complement of mycobacterial DNA replication and repair proteins (Mizrahi and Huberts, 1996; Rock et al., 2015).

Despite all these challenges, there have been some exciting recent discoveries—for example, the novel DnaN-targeting natural product antibiotic, griselimycin (Kling et al., 2015), and the DnaE inhibitor, nargenicin (Young et al., 2016)—which support the potential for new drug discovery in this area, and also suggest that natural product sources are likely to offer the most promising new agents (Wright, 2017). In the ensuing sections, we discuss the very limited number of validated and experimental anti-TB drugs targeting DNA replication, and provide brief updates on recent progress suggesting the potential to develop additional experimental compounds to inhibit other components of the mycobacterial replication machinery.

### Targeting the mycobacterial Pol III holoenzyme

Although the MOA of antifolate drugs such as sulfamethoxazole, trimethoprim, and *para*-aminosalicylic acid includes depletion of dNTP pools, preventing DNA replication, the impact of these agents on *M. tuberculosis* is polypharmacologic as it also involves inhibition of RNA and protein synthesis (Minato et al., 2015). Therefore, in its strictest sense, there are no anti-TB drugs in clinical use which directly target the DNA biosynthetic machinery in mycobacteria. That said, a handful of very exciting recent studies have established the utility of a number of compounds that prevent DNA synthesis by targeting novel Pol III holoenzyme components in *M. tuberculosis*.

#### Targeting the β clamp, DnaN

Together with the *dnaN*-encoded β clamp, the Pol III^*^ core complex (comprising α and ε subunits only, as *M. tuberculosis* lacks θ) and the clamp loader complex (τ/γ, δ, δ′) form the Pol III holoenzyme (Ditse et al., 2017). Griselimycin, a cyclic peptide antibiotic produced by *Streptomyces* spp., was originally discovered 50 years ago, but was abandoned owing to its unfavorable pharmacologic profile and the availability of other drugs such as RIF (Herrmann et al., 2017). Resurgent interest in neglected antibiotics led a team of investigators from Sanofi and Helmholtz Institute for Pharmaceutical Research Saarland to revisit this compound (Kling et al., 2015) as part of a so-called “rekindling” strategy (Herrmann et al., 2017) to identify potential anti-TB agents. In MOA studies, it was discovered that griselimycin and its metabolically more stable derivative, cyclohexylgriselimycin, bound with very high affinity (equilibrium dissociation constants of 1.0 × 10^−10^ and 2.0 × 10^−10^, respectively) to the *dnaN*-encoded β sliding clamp of *M. tuberculosis*. Importantly, the contrastingly poor binding of these compounds to the human DNA clamp protein, PNCA, results in a very high selectivity index, eliminating any concerns of general cytotoxicity.

X-ray crystallography revealed that griselimycin preferentially binds within a hydrophobic pocket located between domains II and III of DnaN—a target site known to be involved in protein-protein interactions between the β_2_ sliding clamp and other DNA replication and repair proteins such as the Pol IIIα replicative polymerase subunit. As such, griselimycin functions as a protein-protein interaction inhibitor and, notably, is bactericidal against mycobacteria. Moreover, resistance is rare (resistant mutants are identified at a frequency of ~5 × 10^−10^) and incurs a very severe fitness cost: in the non-pathogenic *M. smegmatis* as well as *M. tuberculosis*, griselimycin resistance was shown to depend on sequential amplification of the genomic region containing *dnaN* and the mycobacterial origin of replication (*ori*) site. Perhaps unsurprisingly, this resulted in a severe (slow) growth defect *in vitro*, and did not confer cross-resistance to other antibiotics.

From a drug development perspective, the addition of a cyclohexyl group to Proline-8 in the griselimycin backbone resulted in greater metabolic stability as well as increased lipophilicity, in turn increasing the antimycobacterial potency significantly from an initial minimum inhibitory concentration (MIC) of 1.0 μg/ml for the parental compound to 0.06 μg/ml in the derivative, all under aerobic conditions *in vitro*. The compound was also highly active against intracellular *M. tuberculosis* within macrophages, and in a mouse model—both as a single drug and in combination with the first-line drugs, RIF and PZA. These observations support the potential utility of griselimycin derivatives as anti-TB compounds, possibly to shorten therapeutic duration—though it should be noted that, under anaerobic conditions, the compound exhibited a 100-fold increase in MIC, a result which may have implications for its efficacy as a sterilizing drug. Nevertheless, griselimycin remains an exciting prospect, and is undergoing lead optimization by Sanofi and the TB Alliance (https://www.tballiance.org/portfolio/compound/cyclopeptides). Of further interest, very elegant recent work elucidating the pathway for griselimycin biosynthesis in the producer organism, *Streptomyces* DSM 40835, suggests the feasibility of rational modifications to the core pharmacophore (Lukat et al., 2017), thereby overcoming a common stumbling block in natural product drug development.

#### Targeting the clamp loader complex

*Mycobacterium tuberculosis* possesses a restricted set of four clamp loader subunits: τ/γ, encoded by *dnaX* (though it must be noted that the alternative gene product, γ, has not been observed in mycobacteria), and the δ and δ′ ATPases, encoded by *holA* and *holB*, respectively (Ditse et al., 2017). Consistent with their role in loading the β clamp and co-ordinating leading and lagging strand synthesis, all four subunits are essential in *M. tuberculosis* (DeJesus et al., 2017); however, aside from a number of studies which have identified these components as potentially attractive targets for novel antimycobacterial agents (Anishetty et al., 2005; Kinnings et al., 2010; Xu et al., 2014), there are no reports of any experimental approaches to this effect (Plocinska et al., 2017). For this reason, these proteins are included in the small set of “non-validated, essential targets” identified as worthy of future investigation (Table 3).

**Table 3:** Potential, non-validated, essential *Mtb* targets involved in DNA replication.

**Name**	**Encoding gene^a^**	***In vitro* essentiality**	**Comment**	**References**
PolA/Pol I	*Rv1629*	ED^1^/E^2^	Only the 5′-3′ exonuclease domain is essential; the polymerase domain is dispensable in *Mycobacterium smegmatis* and yields a phenotype of DNA damage hypersensitivity. The exonuclease domain is unable to discriminate against dideoxynucleotide 5′-triphosphates and can be inhibited by chain-terminating nucleotide analogs during DNA synthesis.	Gordhan et al., 1996; Mizrahi and Huberts, 1996
RecO	*Rv2362c*	ED^1^	Involved in DNA repair and RecF-dependent recombination; functions to assemble and disassemble RecA filaments at single-stranded gaps	Mizrahi and Andersen, 1998; Singh et al., 2016
HolA	*Rv2413c*	E^1^^,^^3^	Putative DNA polymerase III δ subunit	
UvrD2	*Rv3198c*	ED^1^/E^2^	Component of nucleotide excision repair and methyl-directed mismatch repair; possesses an essential DNA-dependent ATPase activity linked to DNA translocation and protein displacement, as well as a dispensable helicase activity	Kazarian et al., 2010; Williams et al., 2011
DnaZX	*Rv3721c*	E^1^^,^^2^	Putative DNA polymerase III τ and γ subunits	

*E, Essential; ED, Essential domain*.

a Cole et al. (1998);

1 DeJesus et al. (2017);

2 Griffin et al. (2011);

3 *Xu et al. (2014)*.

#### Targeting the Pol IIIα subunit, DnaE1

*Mycobacterium tuberculosis* encodes a single DNA Pol IIIα subunit, DnaE1, which is essential for chromosomal replication (Boshoff et al., 2003) and, therefore, a potentially attractive target for TB drug discovery (Banos-Mateos et al., 2017). Despite the fact that RNA polymerase represents a very successful therapeutic target in *M. tuberculosis* (Koch et al., 2014) and other pathogens (Ma et al., 2016), and that DNA polymerases have been exploited as therapeutic targets for both anti-viral and anti-cancer drugs (Lange et al., 2011), the number of compounds with demonstrated activity against bacterial replicative polymerases is very low and reduces even further when demonstrated whole-cell activity is applied as a filter (Robinson et al., 2012; van Eijk et al., 2017). There are several classes of compound known to inhibit the PolC-type polymerases: the 6-anilinouracils, which are competitive inhibitors of dGTP binding (Tarantino et al., 1999b; Wright et al., 2005); the guanine inhibitors, which are similar to the 6-anilinouracils in functioning as competitive inhibitors, but which target both PolC and DnaE (Wright et al., 2005; Xu et al., 2011); the non-nucleobase inhibitors, which include the anilinopyrimidinediones (such as 6-anilinouracils, competitive inhibitors of dGTP) (Rose et al., 2006) and the quinazolin-2-ylamino-quinazolin-4-ols (or BisQuinols), whose precise MOA remains to be elucidated but appears to involve competitive binding with the DNA template (Guiles et al., 2009); and, finally, the very recently described dicoumarin, 3,3′-(4-Nitrobenzylidene)-bis-(4-hydroxycoumarin) (Hou et al., 2015). In contrast, finding DnaE1-specific inhibitors has proved much more challenging, with some encouraging exceptions.

Very recent work has identified another natural product, nargenicin A1, as a putative DnaE1 inhibitor (Painter et al., 2015). This compound, a macrolide produced by *Nocardia* sp. ACC18, was shown to be active against both *E. coli* and *S. aureus in vitro* and, importantly, was effective against *S. aureus* in two separate mouse infection models. In *S. aureus*, spontaneous resistance was observed at a very low frequency (~10^−9^), and mapped to *dnaE*. This observation—in combination with *in Vitro* data which confirmed that nargenicin binds to the *S. aureus* DnaE protein in the presence of DNA, thereby inhibiting DNA replication—identified the replicative polymerase as the likely molecular target (Painter et al., 2015). However, the MOA remains to be elucidated definitively: the sole SNP in *dnaE* was not located in the DnaE active site, moreover nargenicin-resistant mutants displayed only low-level resistance (~4-fold over MIC). Although limited literature are available to support the potential antimycobacterial utility of nargenicin, a patent lodged by Merck claims that the compound is bactericidal against *M. tuberculosis* and, on that basis, under development as potential anti-TB agent (Young et al., 2016). It is assumed, therefore, that ongoing work aims to determine whether DnaE1 is the molecular target in *M. tuberculosis* and, furthermore, whether the bacillus is able to develop resistance—and at what cost to replicative fitness.

Compound 251D, a hybrid molecule comprising 6-(3-ethyl-4-methylanilino)uracil and fluoroquinolone moieties is another bacterial Pol IIIα inhibitor that has been identified as worthy of investigation as a potential anti-mycobacterial agent. Whether it will prove efficacious though is unclear: the target of 251D is the PolC-type replicative polymerase (Butler et al., 2007), most commonly found in low-GC Gram-positive bacteria (Timinskas et al., 2014). As noted above, *M. tuberculosis* encodes only the *dnaE*-type, which is found in both Gram-positive and Gram-negatives. Therefore, while bioinformatic analyses have predicted that the compound might be capable of docking with DnaE1, these studies utilized a model based on the replicative subunit from the Gram-negative *Thermus aquaticus* (Chhabra et al., 2011); inhibition of the mycobacterial DnaE1 *in vitro* is still to be demonstrated, so too is the activity in whole-cell assays. As noted elsewhere (Plocinska et al., 2017), an attraction of this type of hybrid compound is the potential to target both DNA gyrase and Pol IIIα with a single molecule, thereby limiting the potential for resistance development in drug-susceptible cases and retaining activity against fluoroquinolone-resistant isolates in drug-resistant TB.

### Targeting the mycobacterial primosome

Together with nine other proteins (namely, Pol IIIα, the β_2_ sliding clamp, ε proofreading subunit, τ, δ, and δ′, DnaA, DNA ligase, and Pol I), the DnaB helicase, DnaG primase, and SSB constitute the basic replication module that is found across almost all sequenced bacterial genomes (McHenry, 2011; Robinson et al., 2012). DnaB and DnaG form the helicase-primase complex which, in combination with the PriA helicase loader, functions as the mycobacterial primosome: there are no identifiable mycobacterial homologs of DnaC, DnaT, PriB, or PriC. As core proteins, these represent compelling drug targets and, while much further work is required, some recent progress (summarized below) suggests that a validated clinical candidate targeting different components of the primosome is a genuine possibility.

Within any cell, ssDNA generated during DNA replication (as well as other processes, including exposure to genotoxic stress) is vulnerable to damage and prone to form secondary structures that can restrict DNA metabolic processes with potentially lethal consequences. SSB proteins have evolved to protect ssDNA, and so are essential to bacillary viability during normal replication as well as under DNA damaging conditions. Several recent high-throughput screens have been successful in identifying small-molecule inhibitors of SSB-protein interactions (Lu et al., 2010; Marceau et al., 2013; Glanzer et al., 2016). These include an attempt to identify inhibitors of SSB that might disrupt both DNA replication and SOS-mediated resistance pathways within Gram-positive and Gram-negative bacteria (Glanzer et al., 2016): following *in vitro* screening, six molecules were identified which successfully inhibited a broad range of bacterial SSBs, with a further four exhibiting species-specific activity—thereby establishing the potential for both broad-spectrum and species-targeted use. Notably, five of the six compounds were found to have whole-cell activity against a variety of the tested species, of which a single compound, 9-hydroxyphenylfluoron, was associated with minimal activity against the human SSB homolog. While the potential utility of this approach remains to be determined for *M. tuberculosis*, these results suggest the value of investigating SSB as novel anti-mycobacterial target.

An analogous approach sought to identify compounds that specifically target the eight highly-conserved residues at the C-terminus of *Klebsiella pneumonia* SSB with the objective of inhibiting interactions between SSB and other proteins (Voter et al., 2017). Using the interaction between SSB and the essential protein helicase, PriA, as basis for a high-throughput screen of more than 72,000 compounds, this study aimed to identify small molecules capable of inhibiting SSB interactions. Seven SSB-PriA interaction inhibitors were found to bind to SSB, with a further two binding PriA, all with IC_50_ values below 40 μM. No data were presented on the activity (or lack thereof) of these compounds in whole-cell assays; however, this work reinforces a common theme which suggests that protein-protein interaction inhibitors may be of specific value in inhibiting the large complex of proteins which enables DNA replication. In a similar vein, two potential PriA inhibitors, kaempferol and myricetin, were shown to inhibit the ATP hydrolysis activity of *S. aureus* PriA *in vitro* (Huang et al., 2015). While these compounds were also not validated in whole-cell assays, they too represent encouraging steps in the effort to identify antibiotics that target primosome proteins and, importantly, provide useful insight into the isolation of tractable pharmacophores for optimization against the mycobacterial homologs as part of rational structure-activity relationship (SAR) efforts.

The DnaG primase synthesizes primers for lagging strand Okazaki fragments. An early study investigating plant-derived natural products discovered two phenolic monosaccharides from *Polygonum cuspidatum* with low micromolar IC_50_ values against *E. coli* DnaG (Hegde et al., 2004). Similarly, another molecule from *Penicillium verrucosum* was shown to inhibit *E. coli* primase activity in biochemical assays (Chu et al., 2003). However, whole-cell activity was attainable only in a mutant *E. coli* strain deficient in the lipopolysaccharide layer of the cell wall as well as the AcrAB efflux system, reinforcing the potential obstacles associated with permeation and efflux as part of antibiotic discovery. This is echoed in another study which identified two classes of compounds with efficacy against *E. coli* DnaG *in vitro* and in efflux pump-deficient whole-cell assays (Agarwal et al., 2007). In that case, *in vitro* and whole-cell activity analyses of related pyrido-thieno-pyrimidines and benzo-pyrimido-furans identified numerous hits with attractive IC_50_ and MIC values, again suggesting the potential to identify novel primase inhibitors as possible anti-mycobacterial agents. In this context, it is worth noting the “natural validation” of DnaG as a suitable target for inhibiting replication: in many bacteria including *M. tuberculosis*, endogenous production of guanosine tetra- and penta-phosphate, (p)ppGpp, as part of the stringent response prevents the function of the replicative primase, curtailing bacterial growth (Maciąg et al., 2010).

The interaction of primase with the ssDNA template is facilitated by the replicative DNA helicase encoded by DnaB, another essential protein in *M. tuberculosis* (Sassetti et al., 2003; DeJesus et al., 2017). A number of flavonols have been shown to inhibit DnaB function in other bacteria (Griep et al., 2007; Lin and Huang, 2012), though no reports exist regarding the activity of these (or other) compounds in *M. tuberculosis*. Unusually, DnaB is among five mycobacterial proteins that contain inteins, two others of which are also involved in DNA replication: GyrA and RecA. This observation recently prompted the interesting proposal from Dziadek and colleagues (Plocinska et al., 2017) to block the protein splicing machinery as part of a polypharmacologic approach that would prevent activation of these intein-containing proteins, potentially disrupting multiple pathways simultaneously.

### Targeting DNA unwinding: DNA gyrase and DNA topoisomerase

Replication of the chromosomal DNA requires controlled alterations of the DNA topology to ensure processive synthesis while limiting the stresses imposed by negative supercoiling and concatenation of the double-stranded DNA molecule. The type II topoisomerase, DNA gyrase, functions to relieve torsional strain by introducing transient double-strand DNA (dsDNA) breaks which generate negative supercoils in the bacterial chromosome. Unlike those bacteria which rely on two type II topoisomerase enzymes—DNA gyrase and TopoIV—to accomplish these tasks, *M. tuberculosis* employs only a GyrA_2_B_2_ gyrase comprising *gyrA*-encoded supercoiling subunits and *gyrB*-encoded ATPase proteins. As a drug target, DNA gyrase represents one of the most successful in antibiotic history, primarily of the fluoroquinolones which have been used to treat both Gram-negative and Gram-positive bacterial pathogens. A series of chemical scaffolds has been employed in developing successive generations of fluoroquinolones, all of which function as topoisomerase II poisons, stabilizing the cleaved DNA-topoisomerase II complex and so resulting in a large number of double-stranded DNA breaks within the replicating bacillus which are thought to overwhelm the repair machinery, triggering a cascade of events that results in bacterial death (Dwyer et al., 2015). Fluoroquinolones are currently used as second-line anti-TB agents; however, the imperative to reduce the duration of therapy has seen several large clinical trials of novel combination regimens comprising a fluoroquinolone as frontline agent (Gillespie et al., 2014; Jindani et al., 2014; Merle et al., 2014). Although unsuccessful, these trials yielded valuable lessons about the types of preclinical data which might better inform the design of new therapies (Warner and Mizrahi, 2014), as well as the potentially critical role of drug distribution and lesion penetration in ensuring efficacy (Prideaux et al., 2015).

Other classes of gyrase inhibitors include the aminocoumarins, such as novobiocin, which were the first of many natural products found to act as gyrase inhibitors (Barreiro and Ullán, 2016). Since these compounds preferentially inhibit ATPase (GyrB) function (Lewis et al., 1996), they are less vulnerable to pre-existing resistance against the fluoroquinolones, which generally maps to mutations in *gyrA* (Chopra et al., 2012). Moreover, in contrast to the fluoroquinolones which result in dsDNA breaks and so upregulate the mycobacterial DNA damage response (Gillespie et al., 2005), the risk of aminocoumarin-induced mutagenesis is likely to be lower, especially in *M. tuberculosis* in which exposure to novobiocin does not trigger expression of the SOS regulon (Boshoff et al., 2004). However, the relatively poor penetration of aminocoumarins across cell membranes, their limited solubility, and the development of the synthetic fluoroquinolones have limited the clinical utility of this compound class (Barreiro and Ullán, 2016). In addition, issues with cytotoxicity remain a major hurdle, particularly for TB which requires extended therapeutic duration. Recent progress in the development of novel bacterial topoisomerase inhibitors (NBTIs) targeting DNA gyrase (Grillot et al., 2014; Blanco et al., 2015; Jeankumar et al., 2015a,b; Locher et al., 2015) nevertheless suggests that alternatives to the fluoroquinolones might become available in the future.

In contrast to the Type II enzymes, Type I topoisomerases have been very sparsely explored for antibiotic drug discovery. These enzymes, which cause single-stranded nicks in relaxing the DNA, perform an essential function in remodeling the chromosome for various processes including DNA replication and recombination, RNA transcription, and condensation and therefore represent an attractive target (Tse-Dinh, 2016). For this reason, Sridevi et al. conducted virtual screens of two chemical libraries for the capacity to dock with *M. tuberculosis* TopA (Sridevi et al., 2015). Subsequent *in vitro* verification of the putative hit compounds identified three with activity against purified TopA: amasacrine, tryptanthrin, and hydroxycamptothecin, a derivative of the anticancer topoisomerase inhibitor camptothecin (Wall et al., 1966). The latter hit compound was subsequently modified with terminal hydrophobic moieties to yield a library of fifteen 7-ethyl-10-hydroxycomptothecin derivatives which exhibited activity against both drug-susceptible and XDR *M. tuberculosis*, with MICs as low as 5.92 and 2.95 μM, respectively—a significant improvement over previous TopA inhibitors (Godbole et al., 2014, 2015). Moreover, the XDR isolates exhibited enhanced susceptibility to five of the hydroxycamptothecin derivatives relative to the drug-susceptible strains, suggesting that this might offer an attractive target in these otherwise highly resistant forms. Furthermore, four hydroxycamptothecin derivatives were identified to be more effective at inhibiting the resuscitation of non-replicating persisters in both nutrient starvation as well as oxidative and nitrosative stress models. These results, together with compounds identified in other studies and which still require validation in whole-cell assays (Ravishankar et al., 2015; Sandhaus et al., 2016), highlight the possibility of successfully and specifically inhibiting TopA as a novel therapeutic target for drug-susceptible and drug-resistant TB.

## Targeting other functions in chromosomal replication

During replication, Okazaki fragments are generated which must be joined together by the bacterial NAD^+^-dependent DNA ligase. The enzyme is therefore essential, making it a highly attractive target for drug development. Inhibition of purified *M. tuberculosis* LigA has been reported numerous times (Gong et al., 2004; Srivastava et al., 2005a,b); however, very few compounds have been shown to exhibit whole-cell, micromolar-range activity against *M. tuberculosis*. Following the high-throughput, *in silico* screening of potential LigA inhibitors, Korycka-Machala et al. identified pinafide and mitonafide as attractive inhibitors of *Mtb* growth *in vitro* (Korycka-Machala et al., 2017). Both compounds exhibited an MIC of 25 μM in 7H9 liquid media and half maximal inhibitory concentration (IC_50_) of 50 μM. In addition, the *in vitro* analysis of LigA inhibition suggested that the two compounds failed to inhibit T_4_ ATP-dependent DNA ligase effectively and, therefore, had specificity for NAD^+^-dependent DNA ligase, which is not utilized by eukaryotes. Although preliminary, these results hold great promise for the development of similar compounds or analogs capable of inhibiting *Mtb* growth at low-micromolar concentrations *in vivo* through the inhibition of LigA.

### Proof-of-concept targets from other bacterial systems

#### Sporulation protein SirA and the SocB toxin

Further insight into the inhibition of essential DNA replicative pathways can be obtained from natural phenomena which characterize normal bacterial physiology. Both DnaA and DnaN have been shown to be inhibited by bacterial-derived molecules, including a sporulation protein in *B. subtilis* and a toxin-antitoxin (TA) system found in *Caulobacter crescentus*. In the first example, the interaction of the sporulation protein SirA with domain I of DnaA prevents the replication initiator protein from binding to the origin of replication during the start of sporulation of *B. subtilis*, effectively inhibiting DNA replication initiation (Rahn-Lee et al., 2011). As noted above, DnaA is essential for initiation of DNA replication, with domain I of DnaA being required for interactions between DnaA monomers and other proteins, such as the essential *dnaB*-encoded helicase (Seitz et al., 2000; Abe et al., 2007). This domain of DnaA is thus an attractive target for therapeutic intervention.

In a further example, the toxin component of the atypical SocAB TA system in *C. crescentus* was found to inhibit DNA elongation through an interaction with the *dnaN*-encoded β sliding clamp (Aakre et al., 2013). Notably, mutations conferring resistance to SocB mapped to the hydrophobic Pol III-binding domain of DnaN, indicating a similar binding site to the previously mentioned antibiotic, griselimycin. The mechanism of resistance is different, though, and so cross-resistance is unlikely. In summary, these examples further validate the inhibition of novel DNA replication components and can potentially be used as a basis for the rational design of synthetic inhibitors against *M. tuberculosis* DnaA and DnaN in the future.

## Other mycobacterial DNA replication and repair functions

**Figure 1:**
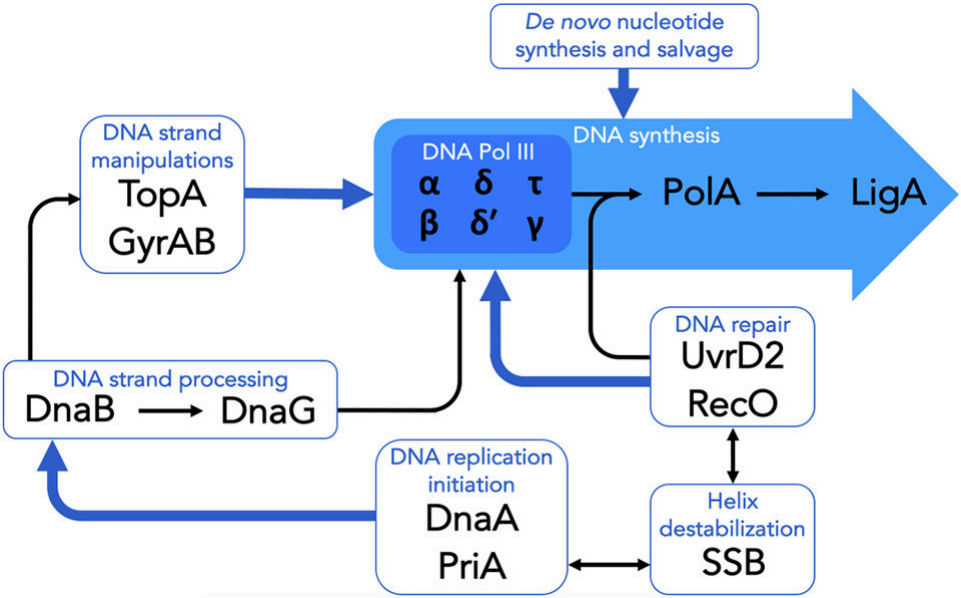
Essential components of DNA replication and repair in *M. tuberculosis*. The schematic highlights the essential DNA replication and repair functions which are targeted by existing clinical or experimental drugs, as well as those which have been identified as potential targets for the development of novel antimycobacterial compounds. See text for details.

In addition to the specialist DNA replication proteins detailed above (and see Figure 1), *M. tuberculosis* encodes a number of other DNA metabolic functions which are essential for cellular viability. For some of these, the potential to yield novel drugs and drug targets is compelling, and includes pathways and enzymes required for *de novo* synthesis, salvage, and recycling of dNTPs for incorporation in newly synthesized DNA, as well as during repair. A detailed discussion is beyond the scope of this review, however some examples include the mycobacterial ribonucleotide reductase (Nurbo et al., 2013; Bueno et al., 2014; Karlsson et al., 2015), thymidylate synthase (Kogler et al., 2011; Fivian-Hughes et al., 2012; Singh et al., 2015), and inosine monophosphate dehydrogenase (Park et al., 2017; Singh et al., 2017) enzymes. Notably, the roles of these and other related proteins in maintaining nucleotide homeostasis within the mycobacterial cell suggests the potential to inhibit replication and repair functions at multiple stages and, moreover, raises the possibility of disrupting indirectly other macromolecular pathways such as RNA transcription and cell wall biosynthesis owing to their convergence on many common metabolic precursors and intermediates (Singh et al., 2015, 2017).

A deeper analysis of other specialist DNA replication and repair functions reveals several more candidates such as Pol I, RecO, and UvrD2, all of which either are essential or contain essential domains (Table 3; DeJesus et al., 2017). For some of these, their perceived potential as novel drug targets requires further validation. PolA/Pol I is a DNA-dependent polymerase that possesses bi-directional exonuclease activity. Previous work identified the 5′-3′ exonuclease domain of PolA as essential to the growth of *M. tuberculosis*; furthermore, while the polymerase domain was shown to be dispensable in *M. smegmatis*, deficiency in this function was associated with DNA damage hypersensitivity (Gordhan et al., 1996; Mizrahi and Huberts, 1996). Importantly, the exonuclease domain was determined incapable of discriminating against dideoxynucleotide 5′-triphosphates, and could be inhibited by chain-terminating nucleotide analogs during DNA synthesis, suggesting the druggability of the target (Mizrahi and Huberts, 1996). Moreover, the involvement of Pol I in DNA damage tolerance has identified this protein as potential target for antimutagenesis agents (Plocinska et al., 2017), as explored further below.

*Mycobacterium tuberculosis* Rv2362c exhibits 28% identity with *S. typhimurium* RecO (Mizrahi and Andersen, 1998), a protein required in DNA repair and RecF-dependent recombination and which functions to assemble and disassemble RecA filaments at single-stranded gaps. Recently, it was reported that *Rv2362c* contains a domain that is essential for *M. tuberculosis* growth (DeJesus et al., 2017), indicating potential of targeting the under-investigated protein with therapeutic compounds in the future. Similarly, UvrD2—a component of nucleotide excision repair and methyl-directed mismatch repair pathways—is another mycobacterial protein containing an essential DNA-dependent ATPase activity implicated in DNA translocation and protein displacement, as well as a dispensable helicase activity (Kazarian et al., 2010; Williams et al., 2011). Although no compounds have been reported to inhibit either of these proteins, their implication in essential replication functions appears to warrant further investigation.

### Targeting mutagenesis

The notion of developing “anti-evolution” drugs to prevent the function of mutagenic repair pathways in *M. tuberculosis* has been discussed previously (Warner, 2010). This strategy seems likely to be especially appropriate for *M. tuberculosis* as adaptive evolution of this organism depends solely on chromosomal rearrangements and point mutations, and all drug resistance arises through spontaneous mutations in target or complementary genes (Galagan, 2014). These factors suggest that inducible mutagenic mechanisms—such as the *imuA*'-*imuB/dnaE2* mycobacterial mutasome (Warner et al., 2010)—might drive the evolution of *M. tuberculosis* within its host. The limited distribution of ImuA′ and ImuB among sequenced bacterial genomes therefore identifies the mutasome as a compelling target for limiting drug resistance. In some ways, this strategy is analogous to targeting virulence factors (Liu et al., 2008) and assumes that the selective pressure to mutate to antibiotic resistance is not as great where the pathway is essential for pathogenesis but not survival (Clatworthy et al., 2007). Moreover, inhibiting mutagenesis should be effective in immune compromised individuals, and might facilitate clinical trials by identifying compounds that could supplement existing regimens without compromising efficacy.

To this end, several approaches appear worth pursuing: in the first, recent evidence suggests that selective inhibition of DnaE2 by anilinouracils might be possible (Jadaun et al., 2015), provided structural data are available to enable rational identification of compounds which target this alternative α subunit, and not the replicative DnaE1, the structure of which was recently elucidated (Banos-Mateos et al., 2017). For this reason, nargenicin may not be appropriate, though dual targeting of both DnaE proteins might nevertheless represent a profitable strategy. Secondly, targeting the PHP domain exonuclease of DnaE1 provides another attractive option as inactivation of this domain was found to render *M. smegmatis* hypersensitive to the chain-terminating adenosine analog, ara-A (Rock et al., 2015). The recent determination of the structure of the PHP domain, which lacks a human homolog, has created an opportunity for structure-guided design of inhibitors against this exonuclease (Banos-Mateos et al., 2017). In the third approach, the identification of griselimycin supports the potential for developing novel protein-protein interaction inhibitors designed to disrupt mutasome function. Further work is underway in our laboratory to elucidate the molecular interactions which are essential to DnaE2-dependent mutagenesis, with genetic evidence indicating that preventing ImuB from functioning as “hub” protein might collapse this pathway (Warner et al., 2010). In conclusion, the possibility of targeting replication and repair mechanisms implicated in the evolution of drug resistance seems a challenge worth tackling: if successful, it is proposed that these compounds might be co-administered with other agents in novel combination therapies designed to protect existing antibiotics.

## Author contribtions

MAR produced the tables and figure, contributed to the main text, and edited the manuscript; DFW co-developed the outline, wrote the main text, and edited the manuscript; and VM developed the outline, wrote the introduction and edited the manuscript.

### Conflict of interest statement

The authors declare that the research was conducted in the absence of any commercial or financial relationships that could be construed as a potential conflict of interest.
